# A case of simultaneous occurrence of acute myeloid leukemia and multiple myeloma

**DOI:** 10.1186/s12885-015-1743-6

**Published:** 2015-10-16

**Authors:** Wang Lu-qun, Li Hao, Li Xiang-xin, Li Fang-lin, Wang Ling-ling, Chen Xue-liang, Hou Ming

**Affiliations:** Departmen of Heamatology, Qilu Hospital, Shandong University, 107# Wenhuaxi Road, Jinan, 250012 P.R. China

**Keywords:** Acute myeloid leukemia, Multiple myeloma, Treatment

## Abstract

**Background:**

Although the occurrence of acute myeloid leukemia (AML) after chemotherapy for multiple myeloma (MM) is common in clinical settings, the simultaneous occurrence of these malignancies in patients without previous exposure to chemotherapy is a rare event. Etiology, disease management, and clinical treatment remain unclear for this particular occurrence. To the best of our knowledge, this study is the first to report a case of simultaneous presentation of AML and MM after exposure to ultraviolet irradiation.

**Case presentation:**

We reported the case of a 73-year-old man (Han Chinese ethnicity) without previous medical history of AML and MM. The morphology and immunology of bone marrow cells confirmed the co-existence of AML and MM. Fluorescent in situ hybridization analysis of immunomagnetically separated abnormal plasma cells showed abnormal expression of the amplified *RB-1*, *TP53*, and *CDKN2C* (1p32). Cytogenetic analysis demonstrated Y chromosome deletion.

After the patient was administered with bortezomib combined with cytarabine + aclarubicin + granulocyte colony-stimulating factor (CAG regimen), and evident curative effects were observed. The patient achieved and maintained complete remission for more than 6 months. Prior to the disease occurrence, the patient had received ultraviolet irradiation for 1 year and was detected with aberrant gene expression of *RB-1*, *TP53*, and *CDKN2C* (1p32). Nevertheless, the correlation of this phenomenon with the etiology of concurrent AML with MM remains unclear.

**Conclusion:**

This study discussed the case of a patient diagnosed with AML concurrent with MM, who has no previous exposure to chemotherapy. This patient was successfully treated by bortezomib combined with CAG regimen. This study provides a basis for clinical treatment guidance for this specific group of patients and for confirmation of the disease etiology.

**Electronic supplementary material:**

The online version of this article (doi:10.1186/s12885-015-1743-6) contains supplementary material, which is available to authorized users.

## Background

The association of acute myeloid leukemia (AML) with multiple myeloma (MM) is described as a complication of chemotherapy but may also occur in the absence of this treatment. The simultaneous occurrence of AML and MM in a patient without previous exposure to chemotherapy is a rare event. Only nine cases of this phenomenon had been reported in the literature until 2003 according to Luca and Almanaseer [1]. These cases of AML concurrent with MM reported from 1989 to 2014 were retrieved from the PUBMED database [2–10].

Three of these cases presented simultaneous occurrence of AML and MM at first diagnosis, even without prior exposure to chemotherapy [1, 3, 6]. Herein we reported a case of simultaneous occurrence of AML and MM in a patient without previous exposure to chemotherapy. This study was approved by the Ethics Committee of the Qilu Hospital of Shandong University. An informed consent form was signed by the patient.

**Fig. 1:**
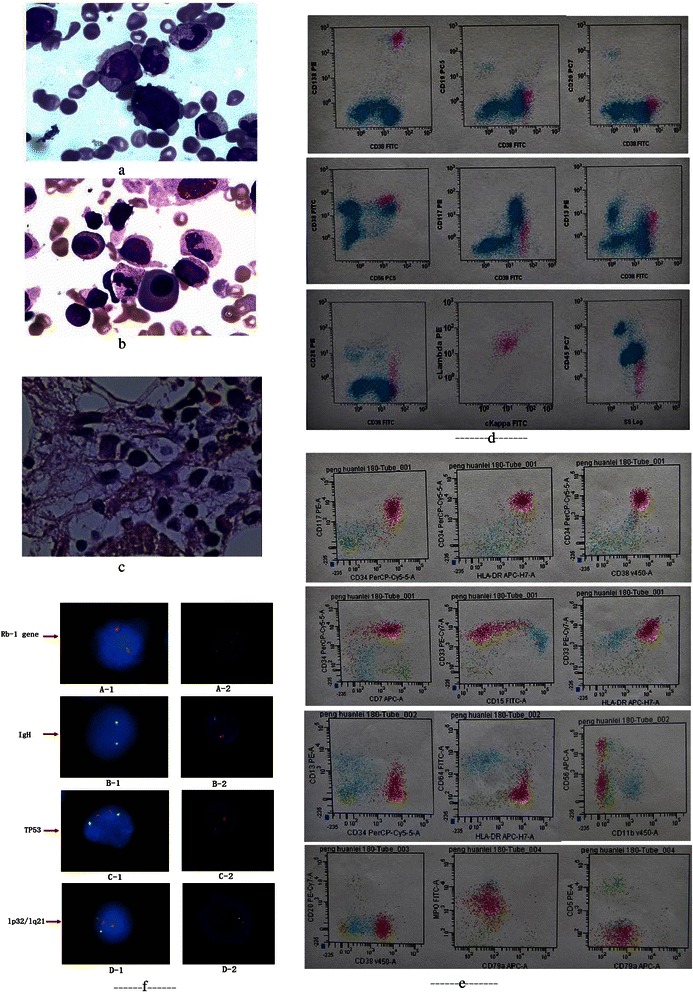
Bone marrow (BM) aspirate smear showed primitive and immature mononuclear cells (**a**: original magnification × 100 under oil) and abnormal plasma cell morphology (**b**: original magnification × 100 under oil). BM trephine biopsy showed increased hyperplasia activity (70 %), widely distributed naive cells, large cell body, abundant cytoplasm, and several irregular nuclei with prominent nucleoli. The percentage of plasma cells increased, and the cells featured special-shaped scattered or clustered distribution with positively stained reticular fibers (**c**: original magnification × 100 under oil). **d**: Flow cytometric immunophenotyping of abnormal plasma cells showed positive CD138, CD38, CD56 and λ expression, and negative CD19 and CD45 expression. **e**: The phenotypic characteristics of malignant myleoid cells showed strong positive CD38 expression, positive CD117, CD34, CD33, HLA-DR, CD56, CD13, and MPO expression, and negative CD5, CD11, CD64, CD20, and CD70 expression. **f**: The gene expression of *RB-1*, *IgH*, *TP53*, and *CDKN2C/CKS1B* as indicated by the results of FISN analysis on immunomagnetically separated abnormal plasma cells. Note: A-1, B-1, C-1, and D-1 for normal bone marrow cells; A-2, B-2, C-2, and D-2 for the patient’s bone marrow cells. Testing of *RB-1* (13q14) by using Vysis and monochrome-labeled probe showed normal 2R signal (A-1) and positive 1R signal characteristics (A-2) (fusion signal showing red color). Testing of *IgH* (14q32) by using Vysis and dual-color separately labeled probe (signal: green for 5′ *IgH* and red for 3′ *IgH*) showed fusion signal with yellow color or green–red overlying color, which represented normal expression of *IgH* in B-1 and B-2. Testing of *TP53* (17p13) by using Vysis and monochrome-labeled probe showed normal 2R signal (C-1) and positive 1R signal characteristics (C-2) (fusion signal showing red color). Testing of *CDKN2C* (1p32)/*CKS1B* (1q21) by using Vysis and dual-color separately labeled *CKS1B*/*CDKN2C* probe (signal: green for *CDKN2C* and red for *CKS1B*) showed normal 2R2G signal and characteristics in D-1 and positive signal for 3R2G 1q21 amplification and 2R1G deletion of 1p32 in D-2

A 73-year-old man without previous medical history bought an ultraviolet irradiation apparatus and received ultraviolet irradiation for 1–2 h daily for 1 year to maintain health and enhance immunity,because he believed that this method can promote local blood circulation, thus benefiting his physical health. (This method is atypical in China.) The patient did not smoke and had no family history of cancer. He had developed progressive fatigue and dizziness for 6 months and presented needle-like subcutaneous hemorrhage on both lower limbs for 1 week. Examination results showed pallor, needle-like subcutaneous hemorrhage, petechiae, sore sternum, and splenomegaly of 1.5 cm under the ribs. The patient had a white blood cell count of 2.1 × 10^9^ per liter, hemoglobin level of 57 g/L, platelet count of 23 × 10^9^ per liter, and erythrocyte sedimentation rate of 156 mm/h. We carried out the detection of serum M-protein by electrophoresis test and the result confirmed the presence of monoclonal immunoglobulin M. Serum immunofixation test revealed a monoclonal IgA/λ band. Quantitative immunoglobulin analysis showed the following contents: IgG 9.22 g/L (NV 7.0–16 g/L); IgA, 14.4 g/L (NV 0.7–4 g/L); IgM 0.33 g/L (NV 0.4–2.3 g/L); IgE1 124.0 (NV 0–100 g/L);β2-MG, 3.09 mg/L (NV 0.7–1.8 mg/L); λ (lambda) light chain, 4.62 g/L (NV 0.9–2.1 g/L); κ (Kappa) light chain, 2.14 g/L (NV 1.7–3.1 g/L), with serum free κ/λ ratio, 0.46 (NV 1.35–2.65). The smear of aspirated bone marrow (BM) cells revealed 45 % myeloblast cells (non-erythroid cells, NEC) and about 17 % highly atypical plasma cells (NEC), as shown in Fig. 1a and b, respectively. X-ray examination revealed no abnormal changes in the patient’s bone. No other signs were observed on the X-ray results.

The results of BM trephine biopsy showed increased hyperplasia activity (70 %), widely distributed naive cells, large cell body, abundant cytoplasm, and several irregular nuclei with prominent nucleoli. The percentage of plasma cell increased, and these cells featured special-shaped scattered or clustered distribution with positively stained reticular fibers (Fig. 1c).

Bone marrow mononuclear (BMM) cells of the patient (one sample) included fluorochrome-conjugated antibodies to the following antigens CD138, CD38 with λ light-chain restriction; another sample of the BMM cells included fluorochrome-conjugated antibodies to the following antigens of CD117, CD33, CD34, HLA-DR, CD15, CD56, CD7, CD17, and MPO. The Cell population classification of some specific antigen with “ + ” and not “-” were detected by using flow cytometry (FACSAriaII, USA). The results of flow cytometric immunophenotyping showed about 13 % atypical plasma cells positive for CD138 and CD38 with λ light-chain restriction, which indicated as multiple myeloma cells (Fig. 1d). Another group cells expressed CD117, CD34, CD33, HLA-DR, CD15, CD56, CD7, CD17 and MPO occupied about 60 % and characterized as malignant myeloid cells (Fig. 1e).

Fluorescent in-situ hybridization (FISH) analysis of immunomagnetically separated abnormal plasma cells showed aberrant expression of the amplified RB-1, TP53, and CDKN2C (1p32) (Fig. 1f). (note: In the present study the hybridized probes of FISH test included RB-1,TP53, Bcr/abL, PML/RARA, AML1/ETO, MLL, FGFR1, CBFB, TET/AML, Bcl-2, MYC, CCND1/IgH; of those negative bio-markers did not listed.) The immune markers of bone marrow myeloid and plasma cells or myeloid cells were determined by flow cytometry. The results showed the positive expression of CD138 in bone marrow plasma cells, whereas CD38, CD117, CD33, CD34, LHA-DR, CD15, CD56, CD7, CD17, and MPO were all positively expressed in bone marrow myeloid cells.

**Fig. 2:**
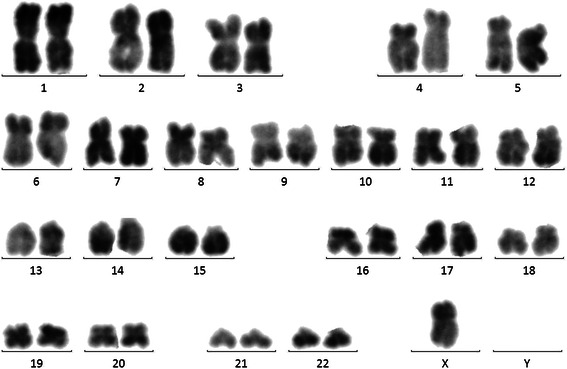
Conventional cytogenetic analysis demonstrated 45 ×, −y [6]

As shown in Fig. 2, conventional cytogenetic analysis demonstrated Y chromosome deletion.

The patient was diagnosed with concurrent AML and MM according to the diagnostic criteria of MM on the international guidelines 2014NCCN (National Comprehensive Cancer Network). He was initially treated with 1.3 mg/m2 bortezomib for days 1, 4, 8, and 11 with 1.3 mg/m^2^ bortezomib, which was combined with the CAG regimen: 10 mg of Acla iv drip for d1–8, 15 mg of Ara-C im q12h for d1–14, and 300 μg of G-CSF ih qd on d1-14. After 2 weeks, bone marrow level was normalized with lower than 5 % residual myeloblast and atypical plasma cells. The patient was treated with the same regimen for three additional cycles and remained in complete stable remission. After treatment, the patient’s dizziness, nausea, fatigue, pallor, needle-like subcutaneous hemorrhage, petechiae, and sore sternum symptoms disappeared; the enlarged spleen of the patient was reduced and did not touch the lower ribs. Laboratory examination showed that Hb was 98 g/L. Quantitative immunoglobulin analysis presented the following contents: IgG, 9.67 g/L (NV 7.0–16 g/L); IgA, 1.35 g/L (NV 0.7–4 g/L); IgM, 0.51 g/L (NV 0.4–2.3 g/L); IgE1, 27.2 (NV 0–100 g/L); β2-MG, 3.51 mg/L (NV 0.7–1.8 mg/L); λ (lambda) light chain, 1.48 g/L (NV 0.9–2.1 g/L); κ (kappa) light chain, 2.22 g/L (NV 1.7–3.1 g/L); and serum-free κ/λ ratio, 1.50 (NV 1.35–2.65). Compared with the results of quantitative immunoglobulin analysis in the diagnosis upon admission, the patient’s IgA and λ levels decreased by 90.62 % and 67.96 %, respectively, with a normal serum-free κ/λ ratio. Immature cells were not found in peripheral blood smear, and the bone marrow normalized with <5 % residual myeloblasts and atypical plasma cells. Cerebrospinal fluid examination did not show any abnormal finding. These results indicated that the patient underwent remission because of the curative effects of both AML and MM. The patient achieved and maintained complete remission for more than 6 months on the last follow-up of March, 20, 2015 (a flow diagram figure shows in Additional file 1).

## Discussion

### Case presentation

Although some reviewers postulated that secondary AML occurs during complete remission of MM after chemotherapy, other scholars hypothesized that myeloma cells can stimulate bone marrow during cell proliferation, this phenomenon may result in subsequent development of a second hematological malignancy, particularly in cases with *Rb-1* deletion [5]. Reports showed that the underlying monoclonal gammopathy of undetermined significance (MGUS) progresses to MM, resulting in the co-existence of MGUS and AML, particularly in elderly patients [9]. The simultaneous development of AML and MM in a patient without previous exposure to chemotherapy is a rare event. The possibility that these two malignancies may originate from common stem cells has not been supported with evidence. Malhotra et al. [11–13] reported 15 cases diagnosed with both Philadelphia chromosome-negative myeloproliferative neoplasms (MPNs) and MGUS or multiple myeloma (MM) at their institute over a period of 5 years (January 2008 to December 2012). Eleven patients with MGUS and two patients with MM had received prior radiation treatment or chemotherapy and then developed MPNs. The two other patients with MM who did not received any cytotoxic treatment developed myelofibrosis. MGUS (Monoclonal gammapathies) denotes the presence of a monoclonal protein without manifesting MM features or other related malignant plasma-cell disorders, such as Waldenstrom macroglobulinemia, primary amyloidosis, B-cell lymphoma, and chronic lymphocytic leukemia [14].

The vast majority of MGUS patients did not present any symptoms. Clinical observations regarding the development from MGUS to MM indicated the absence of symptoms such as anemia, bone destruction, hypercalcemia, and renal function damage; only the serum M protein and the number of bone marrow plasma cells showed changes [15]. In the current study, we report a patient who underwent regular physical examination annually for 5 years and did not manifest any clinical symptoms, such as anemia, bone destruction, hypercalcemia, renal function damage, and abnormal immunoglobulin items, through routine clinical laboratory examinations. The present case was demonstrated myeloid cell malignancy and atypical plasma cells on cytology based on the immune markers of bone marrow myeloid cells and bone marrow plasma cells were determined by flow cytometry. The results showed that the CD138 positive expression of bone marrow plasma cells,and the CD38 strong expresion, the CD117, CD33, CD34 and LHA-DR positive expression, and CD15,CD56, CD17 and MPO part or weak positive expression of bone marrow myeloid cells and FISH analyses of magnetically separated plasma cells. The presence of the M protein in immune fixation electrophoresis supported the diagnosis of concurrent AML and MM without history of chemotherapy except ultraviolet irradiation.

The mechanism of the simultaneous occurrence of AML and MM without exposure to chemotherapy remains unclear. The deletion of RB-1, TP53, and lP32 was associated with the simultaneous occurrence of AML and MM. We speculate that multiple gene mutation or some susceptible genes may be involved in the simultaneous occurrence of both malignancies. Nevertheless, the mechanism underlying the simultaneous occurrence of AML and MM must be further investigated.

Studies have reported that disease management in patients who developed MM focuses on myeloma treatment. Anti-cancer agents, such as thalidomide, lenalidomide, and pomalidomide, demonstrated evident activity in MPN and MM and should be considered in the treatment regimen [16].

The concurrent prognosis of AML and MM remains very poor, and a standard treatment regimen has not been established. Murukutla et al. [10] summarized the therapy experiences of patients in prior reports, which included the use of drugs, such as bortezomib, tipifarnib, cyclophosphamide, vincristine, cytarabine, idarubicin, melphalan, and prednisone. Recently, Kim et al. [8] reported a 51-year-old man who had no past medical history but presented with simultaneous diagnosis of myeloma and AML, which were successfully treated with allogeneic stem cell transplantation. However, these therapy experiences are insufficient to construct a model protocol. The combination of bortezomib with CAG exhibits evident curative effects on elderly patients who are not suitable for allogeneic stem cell transplantation. In the present case, the patient achieved and maintained remission for more than 6 months. This finding may benefit the selection of optimum treatment options for this specific group of patients.

UV radiation is a complete carcinogen, especially for long-term management of children and young adults and in combination with topical or systemic immunosuppressants [17]. We suspect the patient’s malignancy may be related to exposure to the UV radiation, but that no data to proof this hypothesis can be given.

The genetic and molecular biomarkers of a case with simultaneous AML and MM have made considerable progress with the technological developments of flow cytometry and FISH. Before 2003, all case reports of simultaneous presentation of AML and MM performed a type of serum immunofixation test that revealed the types of paraproteins in patients, including IgA, IgA/k, IgG, IgG/k, and IgG/λ; however, few cases conducted the chromosome type test, and the results displayed 46XY and hypodiploidy, as well as chromosomal abnormalities [3, 10]. Luca DC and Almanaseer IY (2003) [1] performed an immunohistochemical test (flow cytometric analysis), which demonstrated myeloblasts with positive expression of Cd14, CD33, and HLA-DR, and a negative expression of CD45 for plasma cells; cytogenetic test showed that the karyotype was monosomy 13. Kim et al. [8] reported a case of simultaneous presentation of AML and MM with k-type paraprotein; immunohistochemical test of the case revealed plasma cells to be positive for CD138 with kappa light chain restriction and myeloblasts to be positive for CD34 and CD117; flow cytometric test confirmed the presence of two distinct neoplastic populations of plasma cells and myeloblasts; fluorescence in situ hybridization (FISH) test revealed a complex chromosomal pattern, with +5, +7, +8, +8q22, +11q23, −13q14, −16q22, +17q13.1, +20q12, and +21q22, and immunoglobulin heavy chain (IgH) rearrangement. The present study showed that the new biomarkers included the abnormal expression levels of the amplified RB-1, TP53, and CDKN2C (1p32) for plasma cells by FISH, as well as the positive or partial expression of CD33, CD15, CD56, CD7, CD17, and MPO for myeloblasts via flow cytometric test.

In the present study, the hybridized probes of FISH test included RB-1, TP53, Bcr/abL, PML/RARA, AML1/ETO, MLL, FGFR1, CBFB, TET/AML, Bcl-2, MYC, and CCND1/JgH. However, gene mutation was not detected for the negative biomarkers by FISH analysis. Some recent reports showed that FLT3 ITD, NPM1, or CEBPA mutation is associated with AML [18–20]. Testing the gene mutation for the negative biomarkers of FISH analysis is the most feasible idea. However, the limitation of this study was the failure to perform the gene mutation test for the negative molecules of FISH test, such as FLT3, ITD, NPM1, and CEBPA.

## Conclusion

A patient without previous exposure to chemotherapy was diagnosed with concurrent AML with MM and successfully treated using bortezomib combined with the CAG regimen. This treatment strategy could be a reasonable option for future cases with similar diagnosis. The findings presented in this case report may particularly benefit patients presented abnormal expression of the amplified *RB-1*, *TP53,* and *CDKN2C* (1p32) and the confirmation of the disease etiology (a cheklist item description shows in Additional file 2).

## Consent section

Written informed consent was obtained from the patient for publication of this case report and any accompanying images. A copy of the written consent is available for review by the editor of this journal.

## Additional files

Additional file 1: **Flow diagram figure.** (DOC 33 kb)
Additional file 2: **CARE Checklist.** (DOC 1.44 mb)

## Abbreviations

AML: Acute myeloid leukemia
MM: Multiple myeloma
BM: Bone marrow
MGUS: Monoclonal gammopathy of undetermined significance
MF: Developed myelofbosis
MPN: Myeloproliferative neoplasms
