# Supplementary material for: A case of simultaneous occurrence of acute myeloid leukemia and multiple myeloma
Source: BMC Cancer. 2015 Oct 16;15:724. doi: 10.1186/s12885-015-1743-6 (PMC4609074; doi:10.1186/s12885-015-1743-6)
Supplement: Additional file 1: — Flow diagram figure. (DOC 33 kb) [file 12885_2015_1743_MOESM1_ESM.doc]

Hospitalization

Diagnosis

Chemotherapy: bortezomib combined with CAG regimen

Suffered with severe respiratory infection

Partial remission with HGB 98g/L, and serum immunofixation test still showed monoclonal IgA/λ

Repeat the treatment every 28 days.

After completed 6 cycles , the patient catched near complete remission with serum immunofixation test showed negative, while serum free light chain test showed positive.

From Sep. 2014, the chemotherapy regimen was repeated every 2 months.

After another three cycles therapy, the patient was kept in near CR conditions. He refused the chemotherapy and hospitalization.

2015.7

The patient died with severe respiratory infection in the local community hospital.

2013．12

2014.1

2014.2

2014.3

2014.9

2015.3
